# 

**DOI:** 10.1192/bjb.2024.125

**Published:** 2025-08

**Authors:** Jane Morris

**Affiliations:** Consultant psychiatrist with NHS Grampian, Aberdeen, UK. Email: jane.morris@nhs.scot

**Figure:**
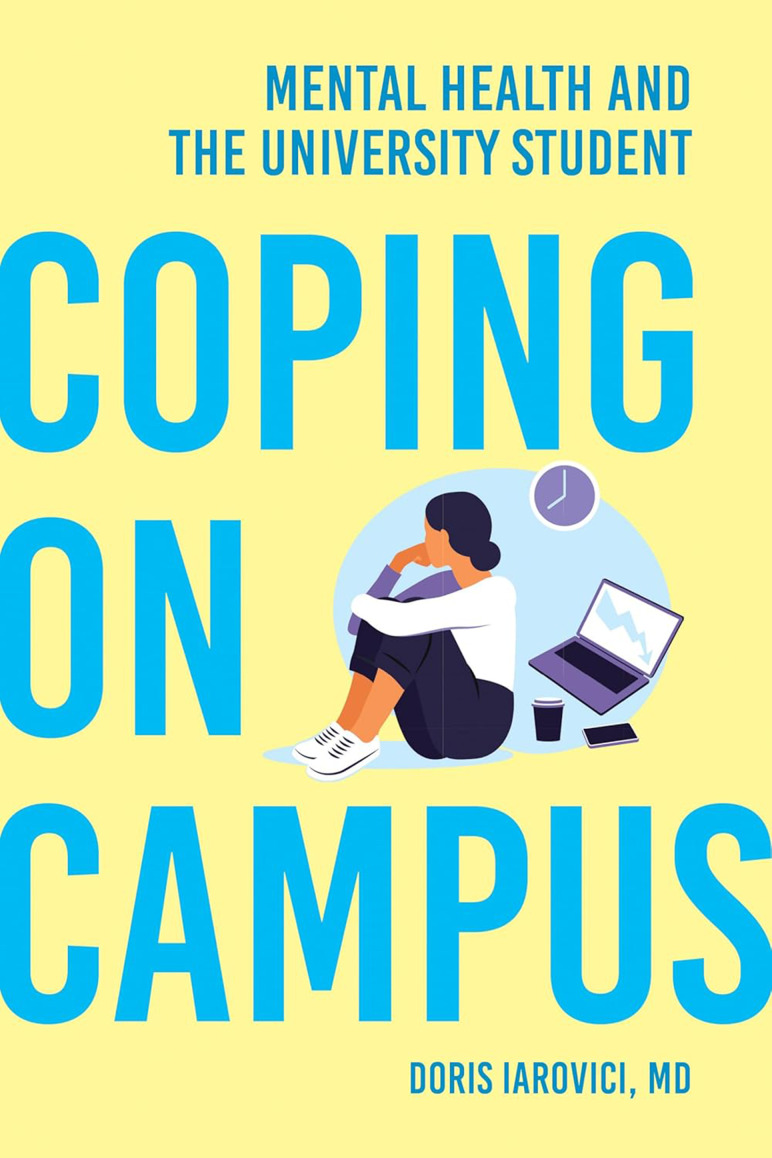

Despite the jaunty title this isn't a self-help paperback to pack into freshers’ luggage. It is a readable, scholarly and clinically relevant account of this important new psychiatric specialty. The 2014 first edition impressed me so much I resolved to write a UK-friendly version (finally published this year).^1^ This disclosure allows you to predict my evaluation of Doris Iarovici's excellent monograph.

The first 50 pages consider current youth mental health and the university environment. The subsequent topic essays could stand alone. Iarovici writes primarily for psychiatrists like herself, who treat university students. The USA already recognises ‘college mental health’, with a substantial psychiatric presence on many campuses and ‘fellowship programmes in child and adolescent psychiatry [which] include emerging adults within their scope of care [ … ] supporting [ … ] transition to college’ (p. 43). We can all heed her advice to show student patients that we are not ‘movie stereotypes’ of ‘the quiet analytical psychiatrist who [ … ] withholds explanations [ … ] They appreciate it when we have a good understanding of the specifics of their campus experience’ (p. 43).

So far, so very good indeed. But outside the USA, this book will not accurately describe that campus experience. Laws relating to minors, mental health and other matters are different, which is problematic when transitions, health service boundaries and bureaucracies are so crucial in the care of our students. American universities – some private, some ‘predominantly Black’, some graduate schools only – have evolved outside a welfare state, and US students face financial as well as bureaucratic obstacles to accessing treatment and purchasing prescribed medications.

Cultural differences are huge. Iarovici's chapter on alcohol tells us ‘most college students are legally underage for consuming alcohol yet most do drink at least occasionally’ (p. 70) . There is ‘campus anxiety’, resulting from mass shootings. Interestingly, despite the characteristic fraternity and sorority structures of US universities, the chapter ‘Loneliness and relationships on campus’ provides an excellent account of international youth culture, covering topics from ‘FOMO’ (fear of missing out) and ‘hookups’ (brief sexual encounters without emotional investment) to ‘ghosting’ (suddenly ending all communication without explanation).

The author shows warmth and empathy towards students but does not extend the same imaginative connection to the adults. She criticises ‘snowplow’ parenting (a style where parents clear all obstacles for their children) and describes the responsibilities of academic and non-academic staff without deep consideration of their own vulnerabilities and mental health needs. Another decade on, I hope to review an even better third edition.
